# Relationships Between Perspective-Taking, Empathic Concern, and Self-rating of Empathy as a Physician Among Medical Students

**DOI:** 10.1007/s40596-019-01114-x

**Published:** 2019-12-23

**Authors:** Jihye Yu, Sukyung Lee, Miran Kim, Kiyoung Lim, Kihong Chang, Mijin Lee

**Affiliations:** Ajou University School of Medicine and Graduate School of Medicine, Suwon, Gyeonggi-do South Korea

**Keywords:** Medical student, Perspective-taking, Empathic concern, Empathy as a physician

## Abstract

**Objective:**

The aim of this study was to ascertain the relationships between perspective-taking, empathic concern, and self-rating of empathy as a physician among medical students.

**Methods:**

This study analyzed the questionnaire responses of 152 medical students enrolled in Ajou University School of Medicine, Suwon, Republic of Korea, in 2018. As measurement instruments, the authors applied the Interpersonal Reactivity Index (IRI) and Korean Student Version of the Jefferson Scale of Physician Empathy (Korean JSPE-S), and then examined participant characteristic variables based on the obtained data and conducted subsequent correlation analyses of subscales, one-way ANOVA, and regression analyses.

**Results:**

Medical students with clinical clerkship experience demonstrated higher levels of perspective-taking and empathy as physicians than did students without experience. Moreover, perspective-taking and empathic concern were significant predictors of medical students’ empathy as physicians in the regression model.

**Conclusions:**

Medical students with higher scores in perspective-taking and empathic concern demonstrated higher levels of perception regarding the necessity and importance of empathy as a physician in patient-physician relationships. Therefore, in actual medical situations with patient-centered therapy, to enhance the levels of physician empathy, medical education should focus on the understanding of other persons’ opinions and interpersonal interactions accompanied by empathic concern.

Communication is an essential factor in a patient-physician relationship [1]. To establish a therapeutic relationship between a patient and a physician, patient-centered communication, which requires physician’s empathy, is needed [2]. Empathy is the ability to understand a patient’s situation, perspective, and feelings, and to communicate with the patient in order to better understand the patient’s perspective and experience [3, 4]. Empathy plays an important role in establishing a positive physician-patient relationship, enhancing patient satisfaction and participation in care, and producing a positive clinical outcome [4–6]. Empathy improves the quality of information obtained from patients, improves the diagnostic ability of physicians, and reduces the incidence of problems such as communication problems and legal litigation [4, 7]. In addition, a physician’s empathy toward patients has been found to affect the outcomes of treatment [8]. A lack or insufficient amount of empathy has been shown to lead to decreased patient satisfaction and has even resulted in adverse medical results due to communication errors [9, 10].

It may be stated that empathy toward patients is a crucial quality that physicians should cultivate. This is especially true in our current era of artificial intelligence. Present and future medical students need to achieve emotional intelligence and skills, which can only be afforded by *Homo sapiens* by communicating and sympathizing with one another, a facility that cannot be fully comprehended or substituted by AI machines. Previous studies have shown that as medical students progress in their education, they exhibit decreasing levels of empathy [3, 11]. The reasons for such decreased empathy in medical students were excessive study overload, lack of time, and an educational environment that emphasizes objective and scientific-based thought and reasoning [3]. Under these circumstances, it is very important that education be systematically conducted to develop and improve the empathy of medical students. To do so, current medical students’ perceptions of empathy in the physician-patient relationship should first be investigated. Hence, this study examines medical students’ empathy as physicians and seeks to ascertain the relationships between individual medical students’ perspective-taking (PT) abilities and levels of empathic concern (EC).

The current study proposed the following questions in accordance with the study aims: First, are there differences among medical students in terms of PT, EC, and self-rating of empathy as a physician by gender, year of study, and clinical practice experience? Second, what is the nature of the relationships between medical students’ PT, EC, and self-rating of empathy as a physician?

## Methods

Our study included medical students enrolled in the first to fourth years of Ajou University School of Medicine, Suwon City, Gyonggi-do Province, Republic of Korea. A questionnaire was sent in June 2018 to 178 students. All students gave consent prior to the provision of questionnaires and responded anonymously. The final number of respondents was 152, after excluding non-responders or inadequate responses.

To measure PT and EC, the two Interpersonal Reactivity Index (IRI) subscales of PT and EC developed by Davis [12] were employed. PT is defined as the tendency to adopt another person’s psychological point of view and measures the level of the cognitive component of empathy. EC is defined as the tendency to feel sympathy and compassion toward another person and may be considered a measure of the affective component of empathy. The current study measure comprised 14 questions, 7 on PT and 7 on EC. For example, a statement that shows PT is, “When I’m upset with someone, I usually try to ‘put myself in his shoes for a while.’” An example of a statement to measure EC is, “When I see people being treated unfairly, I sometimes don’t feel very much pity for them.” Each question was scored on a 5-point Likert-type scale that ranged from *does not describe me well* (1 point) to *describes me well* (5 points), and higher scores reflect higher levels of EC. The values of Cronbach’s *α* for PT and EC were 0.727 and 0.802, respectively, indicating acceptable to good internal consistency.

To measure empathy as a physician, the Korean Student Version of the Jefferson Scale of Physician Empathy (Korean JSPE-S) [13] was employed. This scale comprises a total of 20 questions scored on a 7-point Likert-type scale ranging from 1 = *strongly disagree* to 7 = *strongly agree*. In this study, the JSPE comprised two subscales: 10 p PT and 10 compassionate care and standing in the patient’s shoes (CC/SP) questions. An example of statements showing PT is, “Physicians should try to think like their patients in order to render better care.” CC and SP are assessed through statements such as “Attention to patients’ emotions is not important in history taking” and “It is difficult for a physician to view things from patients’ perspectives.” Higher points indicate a higher degree of empathy as a physician. The value of Cronbach’s *α* for this scale was 0.908.

Descriptive statistical methods were used to determine the distribution of participants by gender, year of study, and clinical practice experience and to ascertain the characteristics of the questionnaire participants. Multiple regression analysis was also conducted to analyze the effects of PT and EC on Korean JSPE-S scores.

## Results

The total number or questionnaire participants was 152, 97 male (63.8%) and 55 female (36.2%). By year of study, 44 (28.9%) were first-year students, 34 (22.4%) second-year students, 35 (23.0%) third-year students, and 39 (25.7%) fourth-year students.

We analyzed the effects of gender, year of study, and clinical practice experience on PT, EC, and JSPE-S scores. The results are shown in Table 1. There was a significant difference by gender with respect to EC such that higher levels of EC were observed among female students than male ones. PT levels were significantly higher in fourth-year than first-year medical students. In addition, a significant difference was demonstrated between PT and self-rating of empathy as a physician by experience of clinical practice.

**Table 1:** Perspective-taking (PT), empathic concern (EC), and Korean JSPE-S scores by student characteristics

Variable		PT	EC	Korean JSPE-S
Gender				
	Male	3.53 (0.56)	3.56 (0.59)	4.97 (0.77)
	Female	3.56 (0.43)	3.81 (0.60)*	5.14 (0.68)
Year of study				
	First-year	3.36 (0.56)	3.51 (0.55)	4.92 (0.73)
	Second-year	3.52 (0.50)	3.62 (0.56)	4.89 (0.69)
	Third-year	3.61 (0.44)	3.86 (0.61)	5.29 (0.75)
	Fourth-year	3.71 (0.50)^†^	3.65 (0.66)	5.09 (0.75)
Clinical practice experience				
	Yes	3.66 (0.47)^‡^	3.75 (0.64)	5.18 (0.76) ^‡^
	No	3.43 (0.54)	3.56 (0.55)	4.91 (0.71)

*p < 0.05 when compared to the male group

^†^*p* < 0.05 when compared to the first-year group

^‡^*p* < 0.05 when compared to the no group

The current study’s regression analysis (Table 2) suggested that PT (*β* = 0.26, *p* < 0.01) and EC (*β* = 0.44, *p* < 0.001) implied a positive effect on medical students’ empathy as a physician.

**Table 2:** Multiple regression analysis of perception of physician’ empathic attitudes

Variable	Korean JSPE-S^a^		
	*B*	SE	*β*
PT^b^	0.38**	0.11	0.26
EC^c^	0.54***	0.09	0.44

^a^*Korean JSPE-S*: Korean Student Version of the Jefferson Scale of Physician Empathy, ^b^*PT*: perspective-taking, ^c^*EC*: empathic concern

**p < 0.01, ***p < 0.001

## Discussion

The following observations were made.

First, we identified the need for further education that nurtures empathy, including PT and EC, to support medical students become more empathic physicians. Our analysis of medical students’ PT, EC, and empathy as physicians showed that there were statistically significant relationships between these three variables. In other words, higher levels of PT and EC in a general relationship were confirmed to play a substantial role in the perception and empathic attitudes that need to be attained by a physician toward patients. One study has shown that educational programs are able to increase medical students’ empathic attitudes [14]. These methods include small-group discussion and role play, as well as educational workshops and seminars, which have been shown to be effective [15, 16]. It will be helpful for empathy training to apply teaching methods such as team-based learning and role play to the medical school curriculum to promote mutual interactions between members and improve communication skills.

Second, clinical education through early clinical exposure is expected to improve the empathy of medical students. This study showed that the empathy as a physician of students who had experienced clinical practice was higher than that in those who had not. This result contrasts with a previous study that reported that students in higher years of study displayed lower levels of empathy [11, 17]. This divergence may be due to the enhancement of medical students’ perception of the patient-physician relationship and their empathic attitudes as a consequence of clinical clerkship experience. In particular, PT abilities, which promote inference of another person’s point of view, differed according to the extent of clinical clerkship experience. This finding suggests that clinical clerkship experience may develop medical students’ abilities to determine a patient’s perspective and attitudes. In the classroom, prior to the clinical practice, it is necessary for the students to be able to experience clinical situations through problem-based learning or team-based learning, using standardized patients.

The significance and implications of the present study are as follows.

Our results indicate that there is a need for medical education that enhances empathy to nurture medical students as physicians who have good empathy with patients. It is imperative to implement education programs and teaching methods to understand the viewpoints of others and to build empathic attention to others.

The limitations of the present study and suggestions for future research are as follows.

The drawback of this investigation was the use of a self-reported questionnaire based on a standardized questionnaire format. It is thought that more objective research perspectives may be obtained through the provision of a specific situation, which would more accurately demonstrate individual students’ empathic attitudes than through standardized questionnaires.

In addition, this study is limited in being a cross-sectional study. While the results suggest that students who experienced clinical practice showed a higher level of empathy and perception than those who did not, further research is needed to clarify the causal relationship and confirm that experience of clinical practice influences empathy. Therefore, we plan to conduct a longitudinal study to track changes in empathy through ongoing surveys each year.
